# Epileptic Encephalopathy and Cerebellar Atrophy Resulting from Compound Heterozygous* CACNA2D2* Variants

**DOI:** 10.1155/2018/6308283

**Published:** 2018-10-15

**Authors:** Kameryn M. Butler, Philip J. Holt, Sarah S. Milla, Cristina da Silva, John J. Alexander, Andrew Escayg

**Affiliations:** ^1^Emory University Department of Human Genetics, Atlanta, GA 30322, USA; ^2^Emory University Department of Pediatrics, Division of Pediatric Neurology, Atlanta, GA 30322, USA; ^3^Emory University Department of Radiology and Imaging Services, Atlanta, GA 30322, USA; ^4^EGL Genetics, Tucker, GA, 30084, USA

## Abstract

*CACNA2D2* encodes an auxiliary subunit of the voltage-dependent calcium channel. To date, there have only been two reports of individuals with early-infantile epileptic encephalopathy due to* CACNA2D2* mutations. In both reports, patients were homozygous for the identified variants. Here, we report a patient with epileptic encephalopathy and cerebellar atrophy who was found to have two novel variants in the* CACNA2D2* gene: c.782C>T (p.Pro261Leu) and c.3137T>C (p.Leu1046Pro), by whole-exome sequencing. The variants were shown to be inherited in* trans* and the unaffected parents were confirmed to be heterozygous carriers. This is the third report of recessive* CACNA2D2* variants associated with disease and the first report of compound heterozygous variants. The clinical description of this new case highlights the phenotypic similarities amongst individuals with* CACNA2D2*-related disease and suggests that* CACNA2D2* should be considered as a differential diagnosis in individuals with cerebellar dysfunction and multiple seizure types that begin in the first year of life.

## 1. Introduction

*CACNA2D2* encodes the alpha-2 and delta-2 auxiliary subunits of the voltage-dependent calcium channel. Expression of the alpha-2-delta-2 (*α*_2_*δ*2) subunit enhances calcium currents produced by the alpha-1 (*α*_1_) pore-forming subunit of the calcium channel by modulating the assembly, trafficking, and localization of the *α*_1_ subunit [1].

To date, there have been two reports of individuals with early-infantile epileptic encephalopathy due to* CACNA2D2* variants [2, 3]. Edvardson and colleagues identified the homozygous* CACNA2D2* missense variant c.3119A>G (p.Leu1040Pro) in three affected siblings from a consanguineous family [2]. Functional analysis in* Xenopus* oocytes revealed that the mutant *α*_2_*δ*2 (L1040P) subunit was unable to enhance currents produced by *α*_1_ channels, suggesting a loss-of-function mechanism [2]. In addition, a homozygous variant of unknown significance in* CELSR3*, which is proximal to* CACNA2D2* on chromosome 3, was also identified in the three affected siblings. In a second report, Pippucci and colleagues identified a homozygous frameshifting* CACNA2D2* variant (c.1295delA, p.Asn432Thrfs*∗*35) in a proband from another consanguineous family [3]. A muscle biopsy from the proband revealed reduced* CACNA2D2* mRNA and protein levels. Notably, this individual also carried a homozygous* CELSR3* missense variant.

Here, we describe an individual with epileptic encephalopathy and compound heterozygous missense variants in* CACNA2D2*. Unlike previous reports, this individual was not homozygous for any* CELSR3* variants.

## 2. Case Presentation

The proband is the male offspring of nonconsanguineous parents born at 39 weeks by cesarean section secondary to fetal distress. Pregnancy was complicated by hypertension and preeclampsia as well as maternal hypothyroidism and migraines. At discharge he was noted to have low muscle tone and unusual eye movements that were jerky with frequent intervals of up gaze. Brain MRIs at one month and five months of age were normal.

**Figure 1:**
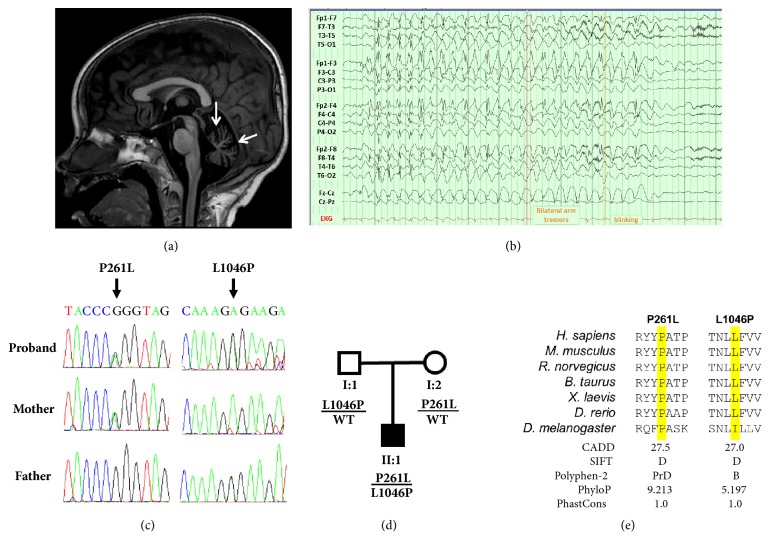
**Clinical and genetic findings from proband with compound heterozygous* CACNA2D2* variants**. (a) Sagittal T1 weighted image from MRI demonstrating cerebellar volume loss (arrows). (b) Awake EEG showing spontaneous generalized 3 Hz spike and wave seizure activity with eye fluttering and low amplitude arm flinches. (c) Sanger sequencing traces showing* CACNA2D2* variants inherited in a compound heterozygous fashion. Note: The* CACNA2D2* gene is encoded by the minus strand of DNA. (d) Pedigree showing the inheritance pattern of the two variants. (e) Species protein alignment showing the evolutionary conservation of the two amino acid positions and prediction scores from* in silico* algorithms. D, deleterious; PrD, probably damaging; B, benign.

At seven months of age, he had onset of seizures with left arm jerking that spread to the left leg and left side of the face with loss of interaction, followed by whole body jerking that lasted 40 minutes. Video EEG over the next 48 hours recorded several additional seizures with multifocal onsets. He was placed on phenobarbital, levetiracetam, and clonazepam. An EEG at eight months of age showed 3-4 Hz slowing. Seizure frequency increased from two seizures per month to 5-10 per day by 14 months of age, at which point he was started on the ketogenic diet. This resulted in six months of seizure freedom; however, seizures recurred. While brain MRIs were repeatedly normal during the first two years of life, an MRI at 29 months of age showed prominence of cerebellar fissures with a normal brainstem, consistent with cerebellar atrophy (**Figure 1(a)**).

At three years and four months of age, he was experiencing five atonic seizures per day and 2-3 seizures per week with apnea and tonic arm flexion. On exam, he could make brief eye contact and smile with a variety of sounds but produced no words. At three years and 11 months of age, seizures consisted of eye fluttering that lasted 2-5 seconds and occurred 1-20 times per day. Treatment with ethosuximide was ineffective. An EEG demonstrated a photoparoxysmal response at 9, 15, 18, and 27 Hz. Several spontaneous (**Figure 1(b)**) and light-induced seizures were recorded by EEG, characterized by 2.5-3 Hz frontally predominant generalized spike and wave discharges accompanied by eye fluttering and arm flinches.

Physical examination at five years of age showed no dysmorphic features. Global hypotonia was noted with unsteady control and mild titubation of the head and torso and moderate ataxia on reaching. He can sit with minimal support but cannot stand or walk. He remains on the ketogenic diet, valproic acid, and clonazepam, with up to 150-day intervals of seizure freedom. Currently, seizures occur only in times of illness, consisting of eye fluttering and behavioral arrest for 3-5 seconds.

Previous biochemical testing was nondiagnostic. Gene panel analysis (Epilepsy and Seizure Disorders Panel), and, later, trio-based whole-exome sequencing (WES), was performed by EGL Genetics. No pathogenic variants were identified by gene panel analysis. During reanalysis of the WES, it was noted that the proband carried two rare missense variants in the* CACNA2D2* gene (p.Pro261Leu and p.Leu1046Pro), which were inherited on separate alleles (**Figures 1(c) and 1(d)**).

Gene panel analysis of 110 genes associated with epilepsy and seizure disorders was performed on DNA from the proband as described previously [4]. Whole-exome sequencing was performed on DNA from the proband and his parents using the SureSelect Clinical Research Exome V1 enrichment kit (Agilent Technologies, Santa Clara, CA). Ninety-seven percent of coding regions were sequenced at >20X coverage. Variant inheritance was confirmed by Sanger sequencing after written consent. The* CACNA2D2* variants were annotated according to RefSeq accessions NM_006030.2 and NP_006021.2. The following primer pairs were used for PCR amplification and sequence analysis of the two variants: 261_F-GTGGCTGAGGGAGGAGAGAA, 261_R-CCTGGATAGGCCGAGAACAG, 1046_F-GTCGCGTTGTAGTCGAAGCA, and 1046_R-CTCGGTAAACGCCTCCTACA. This study was approved by the Institutional Review Board of Emory University.

## 3. Discussion

The two* CACNA2D2* variants identified during WES reanalysis are classified as variants of uncertain significance according to the ACMG-AMP classification criteria [5]; however, there are several lines of evidence to suggest these variants contribute to disease. First, the patient has compound heterozygous variants in a gene associated with autosomal recessive epilepsy and shares many of the clinical features observed for the previously reported patients with homozygous* CACNA2D2* mutations (**Figures 1(c) and 1(d), Table 1**). Second, both variants are extremely rare in the general population; the p.L1046P variant is absent from the gnomAD database, while p.P261L is only observed once out of 245,184 alleles. Third, both variants are located in functional domains of the *α*_2_*δ*2 protein and the affected amino acid residues are evolutionarily conserved (**Figure 1(e)**). Fourth, both variants were predicted to be deleterious by* in silico* algorithms, including CADD and SIFT (**Figure 1(e)**). Finally, one of the proband's variants, p.L1046P, is in close proximity to the previously reported disease variant p.L1040P, which was demonstrated to reduce the function of the *α*_2_*δ*2 protein [2]. Additionally, no other potentially causative variants were identified from WES.

When the clinical presentation of the proband was compared to the two previous reports, we found striking similarities amongst the affected individuals, including seizure onset in the first year of life and hypotonia (**Table 1**) [2, 3]. Additionally, all of the reported patients experience multiple treatment-resistant seizure types without focality and involuntary movements. Of note is the consistent observation of cerebellar atrophy (**Figure 1(a)**), which is also reported in several mouse models of* Cacna2d2* dysfunction [6, 7]. Moreover, it is interesting to note the occurrence of prolonged seizures and photosensitivity for both our patient and the patient described by Pippucci and colleagues [3].

Unlike the two previous reports, the proband in the current study only had one heterozygous missense variant in the* CELSR3* gene (c.5273A>G, p.Gln1758Arg). This variant is common in the general population and has been observed in the homozygous state more than 4,000 times in the gnomAD database, suggesting it is unlikely to contribute to pediatric disease. Furthermore, the observed phenotypic similarities amongst the affected individuals indicate that altered* CACNA2D2* function is the cause of disease.

Because* CACNA2D2*-derived disease is rare, the optimal treatment regime for these individuals is currently unclear; however, the patient reported here did appear to experience improvement while on the ketogenic diet. The mechanism by which* CACNA2D2* dysfunction has been proposed to cause disease involves reduced *α*_2_*δ*2 expression and/or function, leading to reduced *α*_1_ cell surface expression and function [2], which is consistent with the partial clinical overlap observed between* CACNA2D2*- and* CACNA1A*-related disorders. As whole-exome and genome sequencing technologies are applied to individuals with different diseases, it will be interesting to observe if* CACNA2D2* variants are identified in individuals with other disease presentations, such as ataxia or migraine, as has been seen for* CACNA1A.*

In conclusion, we present the third report of a patient with epileptic encephalopathy and cerebellar atrophy due to recessive variants in* CACNA2D2*. Importantly, we present the first case with compound heterozygous* CACNA2D2* variants and no additional candidate disease variants (e.g.,* CELSR3*), further supporting the relationship between pathogenic* CACNA2D2* variants and epileptic encephalopathy with cerebellar atrophy.

## Figures and Tables

**Table 1:** Clinical features of individuals carrying recessive *CACNA2D2* variants.

	This Report	Edvardson *et al*. 2013	Pippucci *et al*. 2013
Genomic position	chr3:50418428	chr3:50402595	chr3:50416390
	chr3:50402577		
cDNA change^a^	c.782C>T	c.3119A>G	c.1295delA
	c.3137T>C		
Protein change^a^	p.Pro261Leu	p.Leu1040Pro	p.Asn432Thrfs*∗*35
	p.Leu1046Pro		
Sex	Male	2 Males, 1 Female	Male
Seizure Onset	7 months	20-60 days	5 months
Epileptic Encephalopathy	+	+	+
Developmental delay	+	+	+
Cerebellar atrophy	+	+	+
Refractory seizures	+	+	+
Seizure types	Absence, atonic, tonic, tonic-clonic	Atonic, clonic, tonic	Absence, clonic, tonic-clonic
EEG	2.5-3 Hz frontally predominant generalized spike and wave discharges	Slow background rhythm with multifocal spikes and slow waves	Multifocal spikes over the right centrotemporal and left parietooccipital regions, slowed background activity
Other features	Status epilepticus, hypotonia, tremor and ataxia, atypical eye movements	Axial hypotonia, choreiform movements, no eye contact	Status epilepticus, axial hypotonia, dyskinetic movements, tremor, no eye contact, facial dysmorphisms, small head, uncoordinated eye movements

^a^ *CACNA2D2* variants annotated according to Refseq NM_006030.2 and NP_006021.2. EEG, electroencephalogram. + indicates presence of feature.
